# Extended *Knee Control* programme lowers weekly hamstring, knee and ankle injury prevalence compared with an adductor strength programme or self-selected injury prevention exercises in adolescent and adult amateur football players: a two-armed cluster-randomised trial with an additional comparison arm

**DOI:** 10.1136/bjsports-2022-105890

**Published:** 2022-10-31

**Authors:** Hanna Lindblom, Sofi Sonesson, Kalle Torvaldsson, Markus Waldén, Martin Hägglund

**Affiliations:** 1 Unit of Physiotherapy, Department of Health, Medicine and Caring Sciences, Linköping University, Linköping, Sweden; 2 Sport Without Injury ProgrammE (SWIPE). Department of Health, Medicine and Caring Sciences, Linköping University, Linköping, Sweden; 3 GHP Ortho & Spine Center Skåne, Malmö, Sweden

**Keywords:** Lower extremity, Preventive Medicine, Soccer, Sporting injuries, Athletic Injuries

## Abstract

**Objective:**

To evaluate the preventive efficacy of an extended version of the *Knee Control* injury prevention exercise programme (IPEP) compared with an adductor strength programme and to a comparison group using a self-selected IPEP in amateur adolescent and adult male and female football players.

**Methods:**

Two-armed cluster-randomised trial with an additional non-randomised arm. All 251 amateur teams (players 14–46 years) in one regional football district were approached. Teams meeting inclusion criteria were randomised to (1) extended *Knee Control* or (2) an adductor strength programme. Teams already using an IPEP were allocated to a comparison group and received no new intervention. Players responded to weekly questionnaires about football exposures and injuries during a 7-month season.

**Results:**

Seventeen teams in the extended *Knee Control*, 12 in the adductor and 17 in the comparison group participated, with 502 players. For the primary outcomes, no difference in injury incidence in three lower-limb injury locations combined (hamstring, knee and ankle) was seen between extended *Knee Control* and the adductor group, whereas extended *Knee Control* had 29% lower incidence than the comparison group (incidence rate ratio 0.71, 95% CI 0.52 to 0.98). No between-group differences in groin injury incidence were seen. The weekly injury prevalence rates in the three lower limb locations combined (hamstring, knee and ankle) were 17% lower (prevalence rate ratio (PRR) 0.83, 95% CI 0.69 to 1.00) and 26% lower (PRR 0.74, 95% CI 0.63 to 0.87) in extended *Knee Control* compared with the adductor and comparison groups, respectively.

**Conclusion:**

No difference in injury incidence was seen between the extended *Knee Control* and the adductor programme whereas extended *Knee Control* reduced injury incidence by nearly one-third compared with a self-selected IPEP. Players in extended *Knee Control* had lower injury prevalence compared with an adductor or self-selected IPEP.

**Trial registration number:**

NCT04272047; Clinical trials.

**WHAT IS ALREADY KNOWN ON THIS TOPIC**

- General injury prevention exercise programmes may reduce injury rates in adolescent and adult football players.
- *The Adductor Strengthening Programme* reduced groin injury rates in subelite male football players in a previous study.
- Effectiveness of injury prevention exercise programmes is generally lower than the preventive efficacy shown in randomised controlled trials.

**WHAT THIS STUDY ADDS**

- Players who performed an extended version of the *Knee Control* programme had approximately one-third lower incidence of injuries to the hamstring, knee or ankle compared with a comparison group that conducted self-selected prevention exercises in adolescent and adult male and female amateur football players.
- Players who used extended *Knee Control* had one-fifth to one-fourth lower prevalence of injuries to the hamstring, knee or ankle compared with the adductor and the comparison group
- Players using the adductor strength programme had no reduction in incidence or prevalence of groin injuries compared with the other groups.
- Players who used extended *Knee Control* had lower incidence of overall time-loss injuries and lower prevalence of substantial injuries than the adductor and comparison group.

**HOW THIS STUDY MIGHT AFFECT RESEARCH, PRACTICE OR POLICY**

- Teams using injury prevention exercise programmes may need more support to ensure an optimal training dosage and progression in order to maximise the preventive effect.
- The extended *Knee Control* programme seems feasible for continued use within male and female amateur adolescent and adult football.
- Continuous support may be needed to ensure that injury prevention exercise programmes are maintained during a full season and over subsequent seasons.

## Introduction

General injury prevention exercise programmes (IPEPs) such as the *Knee Control* programme,^1–3^ the *11+* 4–6 and similar IPEPs^7–10^ focusing on lower limb balance, strength and muscle control, reduce the rate of acute lower extremity injuries in team sports. However, achieving broad-scale effectiveness of IPEPs is challenging.^11^ Coaches often modify programme content or dosage, potentially limiting the preventive effectiveness.^12–14^ We developed an extended version of the *Knee Control* programme, extended *Knee Control*, with the same six main exercises as in the original *Knee Control* programme but with more exercise variations to increase programme fidelity by offering greater variation of exercises, better fit and increased possibility for exercise progressions for football players at various proficiency levels.

Short-focused IPEPs, such as the *Adductor Strengthening Programme*, reduced the rate of groin injuries among male sub-elite football players by 41%,^15^ but the preventive efficacy in other settings is unknown.

The aim was to evaluate preventive efficacy of the extended *Knee Control* programme compared with an adductor strength programme and to a comparison group using a self-selected IPEP in amateur adolescent and adult male and female football players. The preventive efficacy of extended *Knee Control* has not been evaluated before. Our hypotheses were that: (1) extended *Knee Control* would show superior preventive effects on hamstring, knee and ankle injuries and (2) the adductor programme would show superior preventive effects on groin injuries.

## Materials and methods

### Design

This was a two-armed cluster-randomised trial with an additional non-randomised comparison arm. Teams in the randomised groups had not used injury prevention exercises regularly the previous season and were allocated to one of two interventions: Extended *Knee Control* focusing on lower extremity injuries in general, or an adductor programme focusing on groin injuries. The non-randomised comparison group comprised teams that already used an IPEP. Initially, as registered in the study protocol, we intended to include teams that used *Knee Control* 1 2 as comparison group. During inclusion it became apparent that many teams used modifications of the *Knee Control* programme, hence, we broadened the inclusion criteria to better reflect the reality. The study was single blinded with physiotherapists collecting injury data blinded to group allocation. The study covered one 7-month season from March to October/November 2020. The study complied with the declaration of Helsinki and its later amendments. The study has been checked against the CONSORT 2010 checklist, applicable parts of the CONSORT extension for cluster trials^16^ and the CONSERVE 2021 statement.^17^

### Important modifications due to the COVID-19 pandemic

The competitive season was shortened with the start postponed from April to June 2020, and the pre-season correspondingly extended. On 1 April 2020, social distancing was recommended by the Public Health Agency of Sweden and was supported by the Swedish Football Association and the regional football districts. We, therefore, added two exercises to the adductor programme, as described under the Interventions section and table 1.

**Table 1:** Programme descriptions

	Extended *Knee Control*	Adductor programme
Exercises	Running warm-up (5 min) 6 main strengthening and neuromuscular control exercises (10 variations for each) (10–15 min): One-legged knee squat Hamstring strengthening Two-legged knee squat Core strengthening Lunge Jump/landing technique Of these, 44 are individual exercises, including 6 with a resistance band and 16 partner exercises	One exercise out of: Copenhagen adduction, long lever Copenhagen adduction, short lever Side-lying adduction Adductor squeeze (ball between knees, bent legs) Adductor squeeze (ball between feet, straight legs)
Frequency	Every training session throughout the season. Running warm-up before matches.	2–3 times/week pre-season 1 time/week competitive season
Dosage	30–60 s per exercise 2 sets	3–5 to 12–15 repetitions (Copenhagen adduction and Side-lying adduction) or 10 s maximal isometric contractions × 5 repetitions (Adductor squeeze) 1 set
Recommendations about progression	Start at a level that offered sufficient challenge to the players and to progress to more demanding exercises over time. Important that the exercises were done with proper technique before progressing.	Start with Copenhagen adduction long lever. Players unable to perform the exercise with correct technique, prescribed dosage, or who experienced pain >3 on a 0–10 numerical rating scale were recommended to use an easier exercise variant. Adductor squeeze recommended as alternative exercises to avoid close player-to-player contact. Progression mainly through more repetitions during preseason
Equipment	Football, resistance band for some exercises	Football (adductor squeeze)
Setup	Before training, as a warm-up, imbedded in the training, after training or a combination of these	Before or after training

The adductor programme comprised exercises from Harøy *et al*,^15^ the Copenhagen adduction and side-lying adduction, and Hölmich *et al*,^18^ the two adductor squeeze exercises.

### Study population and recruitment

Inclusion criteria for the randomised groups were teams (1) participating in the adolescent or adult 2020 series (male 5th–8th leagues (out of 8 leagues), female 3rd–5th leagues (out of 5 leagues), male and female 16–19 series) in one regional district (Östergötland, Sweden), (2) with at least two scheduled training sessions per week, (3) that had not used an IPEP regularly the previous year. Inclusion criteria 1 and 2 were identical for the comparison group, but these teams had used an IPEP regularly (at least once per week) the previous year and planned to continue in the 2020 season. All players 14 years and older were eligible.

**Figure 1:**
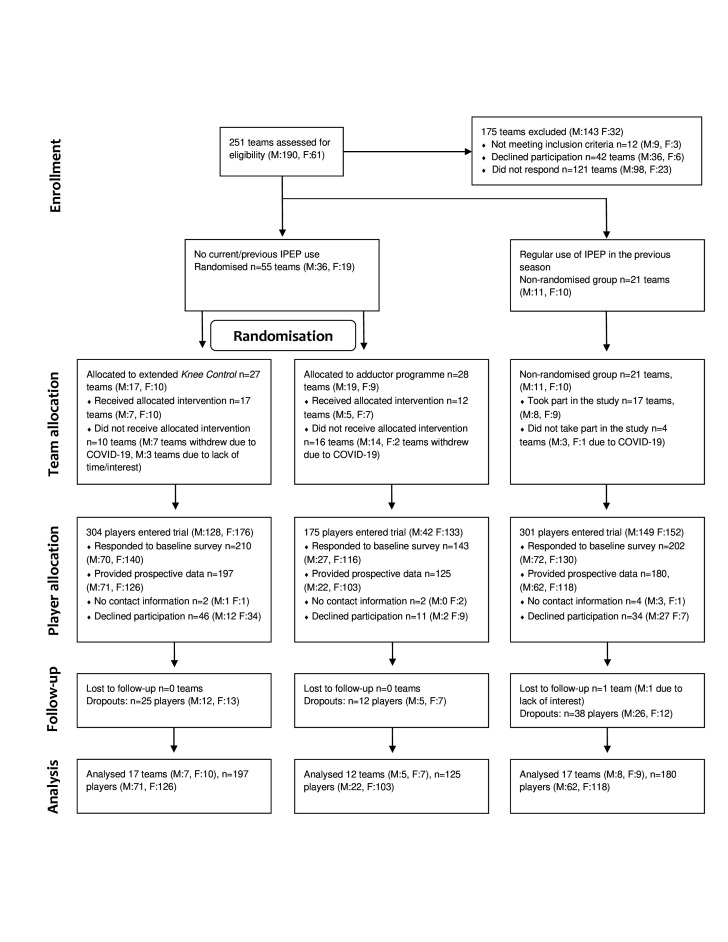
Flow diagram over the inclusion and exclusion of teams and players in the study. F, female; IPEP, injury prevention exercise programme; M, male.

Coaches for eligible teams (n=251) were approached via e-mail and telephone (figure 1) and received oral and written information before accepting participation. Coaches for all participating teams were interviewed by telephone regarding inclusion and exclusion criteria and specifically their use of IPEPs to ascertain the right group allocation (randomised or non-randomised arm). In teams where the coach had accepted participation, players (and guardians for players <15 years) received written information about the study. Response to the questionnaires (baseline and/or weekly) was taken as consent to participate.

### Randomisation

Eligible teams were cluster-randomised with the club as cluster unit, that is, all teams of the same sex in the same club were randomised to the same intervention to minimise risk of contamination. Randomisation was performed by a statistician and stratified by sex and league.

### Interventions

For the randomised groups, coaches and player representatives for each team were invited to practical workshops where the intervention was introduced. Thereafter, coaches led the injury prevention training with their teams. Due to the pandemic, only two workshops were carried out before group gatherings were discouraged. Site visits where the intervention was introduced were made in the remaining teams (n=9), except one team that only received written material and information about the adductor programme via telephone. The workshops were led by two of the main researchers (HL or MH) together with a team of physiotherapists. These physiotherapists also led the practical sessions during site visits. Programme material was offered in written format (folder with pictures and instructions for each exercise) and digitally on a webpage (videos and instructions for each exercise). Teams were recommended to start with their intervention immediately after the workshop/site visit and to use the intervention throughout the season. Coaches were contacted via telephone 1 month after introduction to support training progress.

No workshops or information material were offered to the comparison group. At baseline, coaches from eight teams in the comparison group reported use of the *Knee Control* programme, the others used various self-selected exercises. The teams were instructed to carry on with their usual training throughout the season.

### Extended *Knee Control* programme

Extended *Knee Control* included a running warm-up and six main exercises targeting muscle strength and neuromuscular control with 10 different variations/progression levels for each exercise (table 1, online supplemental material). The programme was built on the *Knee Control* programme (with 30 exercise options)^1^ but had 30 additional exercise options, that is, five extra for each main exercise. Some of the added exercises were easier, to fit younger players, and some were more demanding to fit adult players.

10.1136/bjsports-2022-105890.supp1 Supplementary data

### Adductor strength programme

The adductor programme was initially identical to the *Adductor Strengthening Programme* with one exercise on three levels of difficulty (table 1).^15^ Due to the pandemic, two adductor squeeze exercises^18^ were added as an alternative to avoid close contact between players.

### Data collection

Players responded to baseline and follow-up questionnaires on the use and experience of the IPEPs and adverse events (follow-up questionnaire), most of these data will be presented elsewhere. They also responded to weekly questionnaires about occurrence of injury in any body location based on the Oslo Sports Trauma Research Center (OSTRC) questionnaire (OSTRC-O2),^19^ and exposure to training and matches. Players responded to 1–28 questions per week. When the player reported new injury, additional questions were given regarding nature of the health problem. Injuries were defined in line with the International Olympic Committee consensus statement^20^ and covered any physical complaint injuries (irrespective of need of medical attention or time-loss), sudden-onset and gradual-onset injuries, time-loss injury (injury with reduced participation or absence from football training and/or matches as reported in question 1 of the OSTRC-O2) and medical attention injury (injury where the player sought medical advice or treatment). Substantial injury was defined as an injury with moderate or severe modifications in participation in football training and matches and/or moderate or severe effects on football performance, or inability to participate in football.^19^

One coach per team responded to weekly questionnaires throughout the season and reported number of team matches and training sessions, number of IPEP sessions and timing of IPEP use (before training, during warm-up, imbedded in training, after training, before matches). Players reported which days the IPEP was used in their weekly questionnaires.

All questionnaires were distributed via online software (esMakerNX3 V.3.0) on each Sunday evening with reminders on Tuesday and Thursday the week after. Players who reported time-loss or substantial injury to the groin or hamstrings (sudden-onset or gradual-onset), knee or ankle (sudden-onset) were contacted via telephone by a study physiotherapist who asked about the injury and filled in a standard injury report form. Players could report multiple injuries each week.

### Outcomes

The primary outcomes were any physical complaint injury to either of three lower limb injury locations: hamstring, knee or ankle (hypothesis 1) or to the groin (hypothesis 2). Secondary outcomes were all physical complaint injuries irrespective of location, time-loss injuries, sudden-onset or gradual-onset injuries, substantial and medical attention injuries, adverse events, team and player compliance.

Injury incidence rate (IR) was expressed as the number of new and recurrent injury events per 1000 football hours. All sudden-onset injuries occurring during football training or matches and all gradual-onset injuries regardless of whether symptoms first appeared during football or other activities were included. Weekly injury prevalence rate (PR) was expressed as number of player reports where a player reported new or ongoing injury divided by the total number of eligible player reports that same week.

Compliance to the interventions was described as the weekly dosage based on coach (team compliance) and player weekly reports (player compliance).

### Statistical analyses

An a priori sample size calculation showed that for hypothesis 1, 252 players would be needed based on the assumption that 80% of players would report at least one injury during the season according to the study by Thorborg *et al*, on the *11* and *11+* programmes^21^ a 40% reduction in injury rate in extended *Knee Control* compared with the other groups, and adjusting for clustering effect (estimated design effect 1.28).

For hypothesis 2, 301 players would be needed based on the assumption that 67% of players would report a groin injury during the season according to the study by Harøy *et al*, on the *Adductor Strengthening Programme* 15 and similar injury reduction and design effect as above. With an expected dropout rate of 30%, we aimed to recruit 391 players (approximately 26 teams).

Baseline data are presented descriptively. IR and weekly PR are presented with 95% CI, and incidence and prevalence rate ratios (IRR, PRR) were calculated and compared between all three groups (according to intention-to-treat) using generalised linear models with Poisson distribution, log link and the natural logarithm of total exposure hours or total eligible weeks as offset denominator variables. All data collected from teams and players who dropped out were included in the analyses. Due to uneven sex distribution between groups, all analyses were sex-adjusted. In these calculations, contusions were excluded in line with Waldén *et al*,^1^ since we did not expect that the programmes would prevent these injuries. Sex-separated data were reported descriptively. Absolute rate reduction (ARR) was calculated as a crude estimate of the IR difference for the primary outcome using Poisson distribution and model-based methods to construct 95% CI. Numbers needed to treat (NNT) with 95% CI was calculated as the inverted ARR.^22^ The primary outcomes for incidence and prevalence were tested with regards to cluster effect. Since model fit did not improve, the cluster effect was deemed negligible, and all results are presented without consideration of clustering. IBM SPSS Statistics for Windows (V.27.0. Armonk, New York) was used for all analyses. An experienced statistician was mainly responsible for preparing the databases and analysing data.

### Patient and public involvement

Extended *Knee Control* programme development was informed by a qualitative study with coaches,^13^ and pilot-tested with coaches and players.^23^

## Results

Nineteen clusters with 29 teams were allocated to the randomised groups and received their intervention, 13 clusters with 17 teams were included in the comparison group. In total, 780 players in these 46 teams were eligible, and 502 players (14–46 years) completed the study (table 2). Another 30 teams first accepted participation but withdrew before study commencement without contributing any data. In total, participation rate was 18% of potentially eligible teams.

**Table 2:** Baseline characteristics for included players (n=502)

	Extended *Knee Control* (n=197)	Adductor programme (n=125)	Comparison group (n=180)
Age (years), mean±SD	19.3±5.0	20.5±5.8	20.5±6.3
Sex, n male; female (% male)	71;126 (36.0)	22;103 (17.6)	62;118 (34.4)
Response to baseline questionnaire presented below*	n=178 responses	n=120 responses	n=164 responses
Participating in other sports, n (%)	40 (22.5)	30 (25.0)	33 (20.1)
Estimation of training volume, median (IQR)	5 (1)	5 (1)	5 (1)
Estimation of training load, median (IQR)	5 (1)	5 (1)	5 (1)
Current injury/complaint, ankle, n (%)	24 (13.5)	24 (20.0)	25 (15.2)
Previous injury/complaint, ankle, n (%)	70 (39.3)	37 (30.8)	67 (40.9)
Current injury/complaint, knee, n (%)	28 (15.7)	27 (22.5)	34 (20.7)
Previous injury/complaint, knee, n (%)	74 (41.6)	41 (34.2)	61 (37.2)
Current injury/complaint, hamstrings, n (%)	9 (5.1)	5 (4.2)	13 (7.9)
Previous injury/complaint, hamstrings, n (%)	41 (23.0)	20 (16.7)	31 (18.9)
Current injury/complaint, groin, n (%)	21 (11.8)	6 (5.0)	13 (7.9)
Previous injury/complaint, groin, n (%)	54 (30.3)	38 (31.7)	47 (28.7)

Estimation of training volume and training load was made on a 1–7 Likert scale, where 1 represents low and 7 high-training volume or load (amount of training and intensity of training). Current and previous complaints describe the number of players who, at baseline, indicated current or previous complaints in the location of interest.

*Missing data from 19 players in extended *Knee Control*, 5 players in the adductor group and 16 players in the comparison group who did not respond to the baseline questionnaire.

In total, 6601 weekly player reports were collected. The average weekly response rate was 64.8% (men 55.7%, women 68.1%), 68.0% in extended *Knee Control* (men: 56.1%, women 73.8%), 60.4% in the adductor (men 53.8%, women 61.5%) and 64.4% in the comparison group (men 55.9%, women 67.8%).

### Exposure and injury characteristics

Exposure to training and matches is presented in online supplemental table 1. In total, 458 unique injury events were reported (514 injury locations) in 279 players (online supplemental tables 1,2). Of these 458 injury events, 412 (90%) affected one body location, 39 (8.5%) two and 7 (1.5%) ≥ three locations. Of all injuries, 261 (57%) resulted in time loss.

10.1136/bjsports-2022-105890.supp2 Supplementary data

10.1136/bjsports-2022-105890.supp3 Supplementary data

### Compliance

Team compliance during pre-season was 2.3, 1.7 and 1.9 sessions per week for the extended *Knee Control*, adductor and comparison groups (online supplemental table 1). Corresponding figures for the competitive season (summer break excluded) were 2.1, 0.7 and 2.1 sessions per week. Players in extended *Knee Control* used the IPEP 1.6 times/week on average, the adductor group 1.0 and the comparison group 1.4.

### Intervention effect on the incidence of football injuries

For the primary outcomes, no difference in injury IR in the three lower-limb locations combined (hamstring, knee and ankle) was seen between the extended *Knee Control* and the adductor group, whereas extended *Knee Control* had 29% lower incidence (IRR 0.71, 95% CI 0.52 to 0.98) than the comparison group (table 3). The ARR between extended *Knee Control* and the comparison group was 3.2 (95% CI 0.3 to 6.1) injuries/1000 hours and the NNT was 316 (95% CI 165 to 3620) hours, meaning that to prevent one injury approximately seven players must perform extended *Knee Control* during one season. No differences in groin injury incidence were seen between groups. For secondary outcomes, time-loss injury incidence was 42% and 48% lower in extended *Knee Control* compared with the adductor and comparison groups.

**Table 3:** Injury incidence rates per intervention group and sex-adjusted pair-wise incidence rate ratios between groups

	Extended *Knee Control* (1)	Adductor programme (2)	Comparison group (3)	(1) vs (2)	(1) vs (3)	(2) vs (3)
	IR (95% CI)	IR (95% CI)	IR (95% CI)	IRR (95% CI) p value	IRR (95% CI) p value	IRR (95% CI) p value
**Primary outcomes**						
Three lower-limb locations combined*	7.72 (6.18 to 9.66)	9.36 (7.01 to 12.50)	10.89 (8.72 to 13.59)	0.80 (0.55 to 1.17) p=0.246	0.71 (0.52 to 0.98) p=0.036	0.80 (0.55 to 1.15) p=0.226
Groin	2.01 (1.29 to 3.11)	2.85 (1.69 to 4.81)	2.37 (1.47 to 3.82)	0.58 (0.29 to 1.18) p=0.134	0.83 (0.43 to 1.58) p=0.564	1.20 (0.58 to 2.46) p=0.626
**Secondary outcomes**						
Hamstring	1.60 (0.98 to 2.62)	1.02 (0.42 to 2.45)	2.37 (1.47 to 3.82)	1.45 (0.52 to 4.04) p=0.480	0.67 (0.34 to 1.33) p=0.254	0.41 (0.15 to 1.12) p=0.083
Knee	4.01 (2.94 to 5.47)	3.66 (2.31 to 5.82)	5.02 (3.62 to 6.97)	1.09 (0.62 to 1.92) p=0.766	0.81 (0.51 to 1.27) p=0.351	0.65 (0.37 to 1.15) p=0.140
Ankle	2.11 (1.37 to 3.23)	4.88 (3.27 to 7.29)	4.05 (2.81 to 5.82)	0.42 (0.23 to 0.76) p=0.005	0.53 (0.30 to 0.92) p=0.025	1.13 (0.65 to 1.96) p=0.661
All physical complaints	17.15 (14.76 to 19.92)	23.20 (19.31 to 27.88)	24.14 (20.80 to 28.02)	0.73 (0.58 to 0.93) p=0.012	0.71 (0.58 to 0.88) p=0.002	0.94 (0.74 to 1.19) p=0.602
Sudden-onset	7.72 (6.18 to 9.66)	11.81 (9.13 to 15.27)	11.58 (9.34 to 14.36)	0.63 (0.44 to 0.89) p=0.009	0.67 (0.49 to 0.91) p=0.011	1.03 (0.73 to 1.45) p=0.872
Gradual-onset	9.03 (7.34 to 11.10)	10.79 (8.24 to 14.12)	11.58 (9.34 to 14.36)	0.85 (0.60 to 1.20) p=0.363)	0.78 (0.58 to 1.05) p=0.107	0.87 (0.62 to 1.24) p=0.444
Time-loss	8.12 (6.53 to 10.10)	13.64 (10.73 to 17.33)	15.77 (13.11 to 18.96)	0.58 (0.42 to 0.81) p=0.001	0.52 (0.39 to 0.69) p=0.001	0.85 (0.63 to 1.16) p=0.307
Medical attention	4.41 (3.28 to 5.93)	5.90 (4.10 to 8.49)	5.58 (4.09 to 7.61)	0.69 (0.43 to 1.12) p=0.138	0.79 (0.51 to 1.21) p=0.272	0.97 (0.60 to 1.58) p=0.907

Injury incidence rate is reported per 1000 hours of football play. Incidence rates are unadjusted, whereas incidence rate ratios are adjusted for sex. Total exposure in extended *Knee Control* 9971 hours, adductor 4913 hours, comparison group 7165 hours.

*Injuries to any of the following locations: hamstring, knee or ankle. There were 77, 46 and 78 unique injury events to either of these three lower-limb injury locations, and 20, 14, 17 to the groin, in the extended *Knee Control*, adductor and comparison groups, respectively.

IR, incidence rate; IRR, incidence rate ratio.

### Intervention effect on the prevalence of football injuries

For the primary outcomes, injury PR in the three lower limb locations combined were 17% lower (PRR 0.83, 95% CI 0.69 to 1.00) and 26% lower (PRR 0.74, 95% CI 0.63 to 0.87) in extended *Knee Control* compared with the adductor and comparison groups. The adductor group had higher prevalence of groin injury than both other groups. For secondary outcomes, the prevalence of substantial injuries was 27% and 26% lower in extended *Knee Control* than in the adductor and comparison groups (table 4).

**Table 4:** Weekly prevalence of injuries per intervention group and pair-wise prevalence rate ratios between groups

	Extended *Knee Control* (1)	Adductor programme (2)	Comparison group (3)	(1) vs (2)	(1) vs (3)	(2) vs (3)
	PR (95% CI)	PR (95% CI)	PR (95% CI)	PRR (95% CI) p value	PRR (95% CI) p value	PRR (95% CI) p value
**Primary outcomes**						
Three lower-limb locations combined*	10.87 (9.64 to 12.25)	13.45 (11.67 to 15.50)	14.76 (13.15 to 16.57)	0.83 (0.69 to 1.00) p=0.048	0.74 (0.63 to 0.87) p<0.001)	0.87 (0.72 to 1.05) p=0.143
Groin	3.19 (2.55 to 3.98)	4.08 (3.16 to 5.28)	2.26 (1.68 to 3.03)	0.59 (0.42 to 0.83) p=0.003	1.39 (0.96 to 2.01) p=0.082	2.07 (1.39 to 3.09) p<0.001
**Secondary outcomes**						
Hamstring	1.47 (1.06 to 2.04)	2.25 (1.59 to 3.19)	3.74 (2.97 to 4.71)	0.63 (0.39 to 1.02) p=0.059	0.39 (0.26 to 0.58) p<0.001	0.62 (0.41 to 0.94) p=0.025
Knee	5.96 (5.07 to 7.01)	7.11 (5.85 to 8.64)	6.46 (5.42 to 7.69)	0.90 (0.69 to 1.16) p=0.395	0.93 (0.74 to 1.19) p=0.571	1.03 (0.79 to 1.34) p=0.850
Ankle	3.47 (2.81 to 4.29)	4.58 (3.59 to 5.84)	5.13 (4.21 to 6.24)	0.75 (0.54 to 1.04) p=0.082	0.68 (0.51 to 0.91) p=0.009	0.85 (0.62 to 1.17) p=0.314
All physical complaints	22.26 (20.47 to 24.21)	27.82 (25.20 to 30.70)	28.09 (25.83 to 30.54)	0.81 (0.71 to 0.93) p=0.002	0.80 (0.71 to 0.90) p<0.001	1.00 (0.85 to 1.10) p=0.627
Sudden-onset	9.56 (8.41 to 10.87)	11.06 (9.46 to 12.93)	10.66 (9.31 to 12.21)	0.90 (0.73 to 1.10) p=0.290	0.90 (0.75 to 1.08) p=0.256	1.06 (0.86 to 1.31) p=0.599
Gradual-onset	11.93 (10.64 to 13.38)	15.49 (13.58 to 17.68)	15.63 (13.97 to 17.49)	0.77 (0.64 to 0.92) p=0.004	0.77 (0.65 to 0.90) p=0.001	0.94 (0.79 to 1.12) p=0.497
Substantial	12.21 (10.91 to 13.68)	16.41 (14.43 to 18.66)	16.45 (14.75 to 18.36)	0.73 (0.61 to 0.87) p<0.001	0.74 (0.64 to 0.87) p<0.001	0.99 (0.83 to 1.17) p=0.898

Prevalence rates are unadjusted, whereas prevalence rate ratios are adjusted for sex. Total eligible weeks in extended *Knee Control* 2448, adductor 1420, comparison group 1951 weeks.

*Injuries to any of the following locations: hamstring, knee or ankle.

PR, prevalence rate; PRR, prevalence rate ratio.

### Adverse events

At follow-up, 13 (10.8%), 15 (23.4%) and 21 (20.0%) of the players in the extended *Knee Control*, adductor and comparison groups, who responded to the post season surveys, reported episodes of pain or discomfort when performing injury prevention exercises. Pain intensity during the exercises was rated as 5.0, 3.0 and 3.5 in the respective group on a 0–10 numerical rating scale, where 10 represented the worst imaginable pain.

## Discussion

The principal finding of this cluster-randomised trial was that hypothesis 1 was confirmed; players in extended *Knee Control* had 29% lower injury incidence in the three lower-limb injury locations (hamstring, knee and ankle) than in the comparison group, and 17% and 26% lower weekly injury prevalence than in the adductor and comparison groups. We also found significant injury rate reductions in extended *Knee Control* compared with the other groups for most secondary outcomes including incidence of time-loss injuries (42–48%) and prevalence of substantial injuries (26–27%). Considering the absence of a control group that did not use any IPEP, the injury risk reductions seen in extended *Knee Control* are encouraging. Our comparison group had used injury prevention exercises regularly in the previous year, about half the teams the *Knee Control* programme, and thus represent a ‘best-case real-world injury prevention example’. Our findings are in line with a previous systematic review showing a 39% risk reduction in football teams performing the 11+ programme versus control.^21^

Hypothesis 2 was, however, rejected; there were no between-group differences in groin injury incidence and, surprisingly, the highest prevalence of groin injuries was seen in the adductor group. This is in contrast with Harøy *et al*,^15^ who showed 41% lower risk of hip/groin injury with the *Adductor Strengthening Programme*, and from which we based our groin-focused intervention. The lack of preventive efficacy may be related to low compliance with the programme during the pre-season build-up period. We also had to implement alternative single player exercises due to COVID-19-related restrictions, and while the added adductor squeeze exercises have been included in successful rehabilitation programmes^24^ their preventive efficacy has not been established.^18 25^ Hence, it is difficult to compare results of this intervention to the study using only the *Adductor Strengthening Programme*.^15^ Additionally, whereas Harøy *et al*,^15^ included subelite male players, we included adolescent and adult amateur players of both sexes who may have been less devoted to training, and who had different training status and training tolerance. Challenges with low compliance and maintenance have been reported previously^26^ and we find it reasonable to assume that the lack of efficacy may be related to low fidelity with the programme protocol regarding number of repetitions, training frequency, exercise choice and progression. Additionally, players without groin problems may be more motivated to perform a broader generic programme aimed at overall injury risk reduction, rather than focusing on a specific injury. Hence, in line with adding adductor exercises to the 11+ programme,^27^ it could be valuable to integrate the adductor exercises within extended *Knee Control*, to facilitate regular training routines and player motivation. In fact, we incorporated 10 groin-focused exercises, including the five exercises used in the present study, into a further developed *Knee Control* programme; the *Knee Control+*.

### Strengths and limitations

One major strength of this trial was the active comparison group representing real-world implementation of IPEPs. Other strengths were the use of structured validated self-report weekly questionnaires to players and coaches in combination with physiotherapist-collected data enabling detection of injuries and complaints irrespective of time-loss or medical attention and with cross-validation from different sources.

Some limitations should be mentioned. First, even though we met the a priori decided sample size, the low participation rate and the high pandemic-related dropout of teams and players must be considered. It is likely that the most motivated teams and players entered the study, which should be taken into account when considering compliance and external validity of findings. Participation rate among male players was particularly low and the dropout rendered unequal sex distribution between groups, and we therefore sex-adjusted all analyses. Ideally, programme effects should have been evaluated in men and women separately, but due to the small sample, sex-separated data are only presented descriptively. Second, the prolonged pre-season and shortened competitive season, and matches played without spectators due to the pandemic, limit the possibility of comparing injury rates with other samples. Importantly, the situation was the same within the three study arms and should have negligible impact on comparisons of injury rates. Third, we were forced to adapt the adductor programme due to the pandemic by adding two alternative adductor exercises. This complicates comparisons with studies using the original Adductor Strengthening Programme.^20^ Fourth, we relied on self-reported player data restricted to information about injury location and injury onset whereas specific diagnoses or injury types are unclear. Fifth, 20–25% of players engaged in other sports, most often indoor sports such as floorball and handball where the seasons do not overlap with the football season, but it is unknown how this may affect the players’ overall risk of injury. Sixth, the weekly response rate was rather low (65%) and minor injuries may have passed unnoticed, whereas more severe, long-lasting, injuries were detected. The response rate was similar between groups, and it is unlikely that this affected our injury rate comparisons, and was comparable with previous studies in adolescent athletes (58–66%).^2 28 29^ Seventh, due to the small sample and inaccurate data on playing experience and level, we were not able to analyse results based on player age, playing experience or level.

## Conclusion

Amateur adolescent and adult male and female football players who performed extended *Knee Control* had nearly one-third lower incidence of injury to any of the three lower limb injury locations hamstring, knee and ankle compared with a comparison group conducting self-selected prevention exercises. Prevalence of injuries to the same three locations was one-fifth to one-fourth lower in extended *Knee Control* compared with an adductor strength programme and a comparison group. Lower incidence and prevalence of secondary outcomes, such as time-loss injuries and substantial injuries, were also seen in the extended *Knee Control* group compared with both other groups. In contrast, no preventive effect was seen from the adductor programme on groin injury.

## Data Availability

Data are available upon reasonable request.
