# Supplementary material for: Extended Knee Control programme lowers weekly hamstring, knee and ankle injury prevalence compared with an adductor strength programme or self-selected injury prevention exercises in adolescent and adult amateur football players: a two-armed cluster-randomised trial with an additional comparison arm
Source: Br J Sports Med. 2022 Oct 31;57(2):83–90. doi: 10.1136/bjsports-2022-105890 (PMC9872240; doi:10.1136/bjsports-2022-105890)
Supplement: Supplementary data [file bjsports-2022-105890supp002.pdf]

Supplementary Table 1: Training and match exposure, preventive programme use and descriptive data on injury events

	Extended *Knee Control*			Adductor programme			Comparison group		
	**All players**	**Male**	**Female**	**All players**	**Male**	**Female**	**All players**	**Male**	**Female**
**Exposure and preventive programme use**	**n=197**	**n=71**	**n=126**	**n=125**	**n=22**	**n=103**	**n=180**	**n=62**	**n=118**
Training exposure h per player and week, mean ±SD	3.0 ±2.0	3.4 ±2.3	2.8 ±1.9	2.5 ±1.8	2.1 ±1.9	2.6 ±1.8	2.6 ±2.0	2.9 ±2.2	2.5 ±1.9
Match exposure h per player and week, mean ±SD	0.6 ±0.9	0.5 ±0.8	0.7 ±0.9	0.6 ±0.8	0.5 ±0.9	0.6 ±0.8	0.6 ±0.8	0.6 ±0.8	0.6 ±0.8
Total exposure h per player and week, mean ±SD	3.6 ±2.3	4.0 ±2.6	3.5 ±2.2	3.1 ±2.2	2.6 ±2.2	3.1 ±2.2	3.2 ±2.3	3.5 ±2.6	3.1 ±2.2
Preventive programme use, times per week, mean ±SD	1.6 ±1.2	1.8 ±1.4	1.5 ±1.1	1.0 ±0.9	0.9 ±1.0	1.0 ±0.9	1.4 ±1.2	1.3 ±1.2	1.5 ±1.2
Training exposure season, sum h	8211	2548	5664	4003	441	3563	5874	1601	4274
Match exposure season, sum h	1759	374	1385	910	109	801	1291	317	974
Total exposure season, sum h	9971	2922	7049	4913	550	4363	7165	1918	5248
**Injury events (n=458)**	**n=171**	**n=51**	**n=120**	**n=114**	**n=14**	**n=100**	**n=173**	**n=38**	**n=135**
**Primary outcomes**									
Injury to 3 lower-limb locations combined n (%)*	77 (45.0)	25 (49.0)	52 (43.3)	46 (40.4)	6 (42.9)	40 (40.0)	78 (45.1)	10 (26.3)	68 (50.4)
Groin injury n (%)	20 (11.7)	12 (23.5)	8 (6.7)	14 (12.3)	1 (7.1)	13 (13.0)	17 (9.8)	5 (13.2)	12 (8.9)
**Secondary outcomes**									
All physical complaints n (%)	171 (100)	51 (100)	120 (100)	114 (100)	14 (100)	100 (100)	173 (100)	38 (100)	135 (100)
New injury n (%)†	83 (49.7)	21 (42.9)	62 (52.5)	60 (52.6)	8 (57.1)	52 (52.0)	72 (41.6)	15 (39.5)	57 (42.2)
Re-injury same season n (%)†	29 (17.4)	5 (10.2)	24 (20.3)	21 (18.4)	1 (7.1)	20 (20.0)	34 (19.7)	10 (26.3)	24 (17.8)
Re-injury previous season n (%)†	55 (32.9)	23 (46.9)	32 (27.1)	33 (28.9)	5 (35.7)	28 (28.0)	67 (38.7)	13 (34.2)	54 (40.0)
Time-loss n (%)	81 (47.4)	26 (51.0)	55 (45.8)	67 (58.9)	9 (62.3)	58 (58.0)	113 (65.3)	26 (68.4)	87 (64.4)
Medical attention n (%)‡	44 (25.9)	20 (40.0)	24 (20.0)	29 (25.4)	1 (7.1)	28 (28.0)	40 (23.3)	7 (18.9)	33 (24.4)
Sudden-onset n (%)¶	77 (46.1)	23 (46.0)	54 (46.2)	58 (52.3)	11 (78.6)	47 (48.5)	83 (50.0)	19 (54.3)	64 (48.9)
Gradual-onset n (%)¶	90 (53.9)	27 (54.0)	63 (53.8)	53 (47.7)	3 (21.4)	50 (51.5)	83 (50.0)	16 (45.7)	67 (51.1)

Injury data is presented with contusions excluded. Time-loss was defined based on response to the Oslo Sports Trauma Research Center questionnaire (question 1, equal to reduced participation or inability to participate). *Injuries to any of the following locations: hamstring, knee or ankle. Missing data: †4 players extended *Knee Control*, ‡1 player extended *Knee Control*, 1 comparison group, ¶4 players extended *Knee Control*, 3 adductor, 7 comparison group

Abbreviations: h – hours; min – minutes; SD – standard deviation; wk – week
