# Supplementary material for: Extended Knee Control programme lowers weekly hamstring, knee and ankle injury prevalence compared with an adductor strength programme or self-selected injury prevention exercises in adolescent and adult amateur football players: a two-armed cluster-randomised trial with an additional comparison arm
Source: Br J Sports Med. 2022 Oct 31;57(2):83–90. doi: 10.1136/bjsports-2022-105890 (PMC9872240; doi:10.1136/bjsports-2022-105890)
Supplement: Supplementary data [file bjsports-2022-105890supp003.pdf]

Supplementary Table 2 - Injury locations

	Extended *Knee Control*			Adductor programme			Comparison group		
	**All n (%)**	**Male n (%)**	**Female n (%)**	**All n (%)**	**Male n (%)**	**Female n (%)**	**All n (%)**	**Male n (%)**	**Female n (%)**
**Injury locations (n=514)**	**n=197**	**n=56**	**n=141**	**n=125**	**n=14**	**n=111**	**n=192**	**n=40**	**n=152**
**Head and neck**	**6 (3.0)**	**1 (1.8)**	**5 (3.5)**	**4 (3.2)**	**0 (0.0)**	**4 (3.6)**	**5 (2.6)**	**1 (2.5)**	**4 (2.6)**
Head	3 (1.5)	0 (0.0)	3 (2.1)	3 (2.4)	0 (0.0)	3 (2.7)	2 (1.0)	0 (0.0)	2 (1.3)
Neck	3 (1.5)	1 (1.8)	2 (1.4)	1 (0.8)	0 (0.0)	1 (0.9)	3 (1.6)	1 (2.5)	2 (1.3)
**Upper limb***	**5 (2.5)**	**1 (1.8)**	**4 (2.8)**	**6 (4.8)**	**1 (7.1)**	**5 (4.5)**	**7 (3.6)**	**2 (5.0)**	**5 (3.3)**
Shoulder	2 (1.0)	0 (0.0)	2 (1.4)	3 (2.4)	0 (0.0)	3 (2.7)	3 (1.6)	1 (2.5)	2 (1.3)
Wrist	1 (0.5)	0 (0.0)	1 (0.7)	0 (0.0)	0 (0.0)	0 (0.0)	1 (0.5)	0 (0.0)	1 (0.7)
Hand	2 (1.0)	1 (1.8)	1 (0.7)	3 (2.4)	1 (7.1)	2 (1.8)	3 (1.6)	1 (2.5)	2 (1.3)
**Trunk***	**11 (5.6)**	**2 (3.6)**	**9 (6.4)**	**7 (5.6)**	**0 (0.0)**	**7 (6.3)**	**12 (6.3)**	**5 (12.5)**	**7 (4.6)**
Chest	1 (0.5)	0 (0.0)	1 (0.7)	0 (0.0)	0 (0.0)	0 (0.0)	1 (0.5)	1 (2.5)	0 (0.0)
Thoracic spine	4 (2.0)	0 (0.0)	4 (2.8)	1 (0.8)	0 (0.0)	1 (0.9)	1 (0.5)	1 (2.5)	0 (0.0)
Lumbosacral	6 (3.0)	2 (3.6)	4 (2.8)	6 (4.8)	0 (0.0)	6 (5.4)	10 (5.2)	3 (7.5)	7 (4.6)
**Lower limb**	**175 (88.8)**	**52 (92.9)**	**123 (87.2)**	**101 (80.8)**	**13 (92.9)**	**88 (79.3)**	**164 (85.4)**	**30 (75.0)**	**134 (88.2)**
Groin	20 (10.2)	12 (21.4)	8 (5.7)	14 (11.2)	1 (7.1)	13 (11.7)	17 (8.9)	5 (12.5)	12 (7.9)
Posterior thigh	16 (8.1)	7 (12.5)	9 (6.4)	5 (4.0)	0 (0.0)	5 (4.5)	19 (9.9)	5 (12.5)	14 (9.2)
Anterior thigh	13 (6.6)	3 (5.4)	10 (7.1)	6 (4.8)	1 (7.1)	5 (4.5)	18 (9.4)	5 (12.5)	13 (8.6)
Knee	47 (23.9)	13 (23.2)	34 (24.1)	19 (15.2)	2 (14.3)	17 (15.3)	41 (21.4)	4 (10.0)	37 (24.3)
Lower leg	32 (16.2)	4 (7.1)	28 (19.9)	14 (11.2)	2 (14.3)	12 (10.8)	22 (11.5)	4 (10.0)	18 (11.8)
Ankle	28 (14.2)	7 (12.5)	21 (14.9)	26 (20.8)	4 (28.6)	22 (19.8)	35 (18.2)	5 (12.5)	30 (19.7)
Foot	19 (9.6)	6 (10.7)	13 (9.2)	17 (13.6)	3 (21.4)	14 (12.6)	12 (6.3)	2 (5.0)	10 (6.6)
**Unspecified location**	**0 (0.0)**	**0 (0.0)**	**0 (0.0)**	**7 (5.6)**	**0 (0.0)**	**7 (6.3)**	**4 (2.1)**	**2 (5.0)**	**2 (1.3)**

Injury data is presented with contusions included. Up to three injuries could be reported for each player in a single week.

*No injuries were reported to the upper arm, elbow, forearm or abdomen.
